# Evaluating the impact of common clinical confounders on performance of deep-learning-based sepsis risk assessment

**DOI:** 10.3389/frai.2025.1452471

**Published:** 2025-07-15

**Authors:** Shikha Chaganti, Vivek Singh, Alasdair Edward Gent, Rishikesan Kamaleswaran, Ali Kamen

**Affiliations:** ^1^Siemens Healthineers, Princeton, NJ, United States; ^2^School of Medicine, Duke University, Durham, NC, United States

**Keywords:** deep learning, VAE, autoencoder, Sepsis-3, ASE, sepsis, sepsis confounders, ED

## Abstract

**Introduction:**

Early identification of sepsis in the emergency department using machine learning remains a challenging problem, primarily due to the lack of a gold standard for sepsis diagnosis, the heterogeneity in clinical presentations, and the impact of confounding conditions.

**Methods:**

In this work, we present a deep-learning-based predictive model designed to enable early detection of patients at risk of developing sepsis, using data from the first 24 h of admission. The model is based on routine blood test results commonly performed on patients, including CBC (Complete Blood Count), CMP (Comprehensive Metabolic Panel), lipid panels, vital signs, age, and sex. To address the challenge of label uncertainty as a part of the training process, we explore two different definitions, namely, Sepsis-3 and Adult Sepsis Event. We analyze the advantages and limitations of each in the context of patient clinical parameters and comorbidities. We specifically examine how the quality of the ground truth label influences the performance of the deep learning system and evaluate the effect of a consensus-based approach that incorporates both definitions. We also evaluated the model's performance across sub-cohorts, including patients with confounding comorbidities (such as chronic kidney, liver disease, and coagulation disorders) and those with infections confirmed by billing codes.

**Results:**

Our results show that the consensus-based model identifies at-risk patients in the first 24 h with 83.7% sensitivity, 80% specificity, 36% PPV, 97% NPV, and an AUC of 0.9. Our cohort-wise analysis revealed a high PPV (77%) in infection-confirmed subgroups and a drop in specificity across cohorts with confounding comorbidities (47-70%).

**Discussion:**

This work highlights the limitations of retrospective sepsis definitions and underscores the need for tailored approaches in automated sepsis detection, particularly when dealing with patients with confounding comorbidities.

## Introduction

Early detection of sepsis is critical in an emergency department (ED) setting to identify patients with an elevated risk and treat them promptly. While several machine-learning-based decision support tools have been developed to address this challenge, significant obstacles remain (van der Vegt et al., 2023). A key limitation is that machine learning models usually depend on labeled retrospective data. It is difficult to accurately identify sepsis given the lack of a pathological gold standard, as there is no confirmatory test to diagnose sepsis.

Sepsis is defined as the body's dysregulated response to infection; it may lead to multiple organ failure that can be fatal. Over the years, various guidelines have been developed to identify sepsis based on laboratory values, vital signs, and other clinical data. In 1991, a consensus conference (Bone et al., 1992) characterized sepsis using the Systemic Inflammatory Response Syndrome (SIRS) criteria, which identified sepsis as a presumed infection accompanied by at least two of four clinical signs: fever or hypothermia, tachycardia, tachypnea, and abnormal white blood cell count. However, while SIRS criteria were effective in detecting a generalized inflammatory response, they lacked specificity for sepsis and often included non-infectious conditions. In 2001, another consensus conference (Levy et al., 2003) expanded the SIRS criteria by incorporating additional symptoms and organ dysfunction variables. Under this revised framework, the presence of four or more of the expanded criteria due to a presumed infection was indicative of sepsis. Recognizing the limitations of SIRS-based definitions, a task force of experts in sepsis pathobiology, clinical trials, and epidemiology established a new consensus definition in 2016 (Singer et al., 2016), published in the Journal of the American Medical Association (JAMA). This consensus-based definition is widely known as the Sepsis-3 definition, according to which a change in a Sequential Organ Failure Assessment (SOFA) score of greater than or equal to 2, along with suspicion of infection, was indicative of Sepsis. The SOFA score quantifies the extent of organ dysfunction in six different systems (respiratory, cardiovascular, hepatic, coagulation, renal, and neurological). Organ dysfunction could be due to an infection or existing co-morbid conditions such as chronic kidney disease. Suspicion of infection was determined as those who have received antibiotics and had either a urine or a blood culture within a certain time window (see Materials and methods). Although Sepsis-3 definition provides a guideline to establish a suspicion of infection, several studies have shown that when it is used for automated retrospective assessment of sepsis cases, it has a low positive predictive value (Henning et al., 2017), often identifying patients who have organ dysfunction that is not necessarily due to an infection (Henry et al., 2019; Litell et al., 2021). In response to a lack of a robust definition for retrospective surveillance of sepsis, the CDC funded a consortium to create an alternative set of criteria (Rhee et al., 2019). The criteria for presumed infection are stronger with this definition, which requires a blood culture and at least 4 days of antimicrobial therapy. Organ dysfunction is established by a set of criteria that looks at the use of vasopressors, ventilation, and a major change in lab values of creatinine, bilirubin, and platelets (see Materials and methods).

Over the past two decades, numerous automated sepsis detection studies have relied on the SIRS criteria, combined with organ dysfunction parameters from either the SEP-1 or Sepsis-3 definitions, to identify sepsis cases (Ackermann et al., 2022; van der Vegt et al., 2023; Fleuren et al., 2020; Liu et al., 2025). However, it is important to recognize that these criteria, when used in isolation, cannot definitively confirm the presence of sepsis (Henning et al., 2017). As a result, models trained solely on these definitions may incorporate inaccuracies that compromise their reliability. A notable study by Henry et al. (2019) evaluated multiple approaches for retrospectively identifying sepsis using electronic health record (EHR) data, highlighting the limitations of the SEP-1 and Sepsis-3 criteria. In response, they proposed a modified version of the ASE criteria that excludes patients with comorbidities that could independently contribute to laboratory abnormalities indicative of organ dysfunction. While this exclusion strategy offers some benefits, its effectiveness may be limited, as a substantial proportion of sepsis patients are older adults with preexisting comorbidities (Sabir et al., 2022) or have experienced trauma (Osborn et al., 2004; van der Vegt et al., 2023).

In this paper, we compute both Sepsis-3 and ASE labels on a retrospective dataset of 96,992 patients. We begin by analyzing the performance of models trained with these two different labeling systems. Through an automated chart review, we identify common confounders in sepsis definitions. Using results from the models trained with different labels, along with the insights from the chart review, we create various patient subgroups. We demonstrate that the Sepsis-3 definition is more likely to classify patients with organ dysfunction due to preexisting comorbidities, such as chronic kidney disease or liver cancer, without evidence of infection. We then compare the performance of models with different labels and show that a consensus-based approach, which integrates both labeling criteria, yields more robust model performance. Additionally, we evaluate our models across different subgroups, with confounding conditions and confirmed infections. This approach addresses cohort heterogeneity and allows for the future development of more tailored models that incorporate patient-specific clinical information, ultimately enhancing overall model performance.

In summary, we highlight two key challenges in developing automated sepsis prediction models. First, defining the ground truth for sepsis retrospectively is inherently difficult, leading to low replicability, which complicates model training and evaluation. Second, the heterogeneity of sepsis presentations and the presence of multiple confounders further exacerbate these challenges.

## Materials and methods

Our approach involves training a deep neural network (DNN) called Deep Profiler using a large and heterogeneous cohort of patients. The input data includes a range of lab markers, vital signs, and age, as outlined in Appendix Table 1. The model's output is a calibrated probability (Niculescu-Mizil and Caruana, 2005) indicating the likelihood of a sepsis diagnosis in an emergency department setting.

Training began by assigning a diagnosis label to each patient based on either the Sepsis-3 or ASE criteria. The Sepsis-3 label is assigned to patients who meet two conditions (Singer et al., 2016): (1) there is a suspicion of infection, and (2) there is evidence of organ dysfunction, defined by a change in the Sequential Organ Failure Assessment (SOFA) score, within a 72-h window from the time infection is suspected. Suspicion of infection is indicated by a blood culture order within 24 h of antibiotic administration or followed by antibiotics within 72 h. Organ dysfunction is considered present when there is a change in the SOFA score of greater than or equal to 2 within 48 h before or 24 h after the onset of suspicion. The SOFA score is calculated by aggregating the individual organ function scores as defined by the following:

- Respiratory SOFA (1-4 points as the ratio of PaO2 to FiO2 decreases).
- Cardiovascular SOFA (1-4 points as mean arterial pressure decreases or vasopressor dependence increases).
- Liver SOFA (1-4 points as bilirubin increases).
- Coagulation SOFA (1-4 points as platelets decrease).
- Kidney SOFA (1-4 points as creatinine increases).

We do not compute the nervous system SOFA score, as the Glasgow Coma Scale (GCS) score is not available in the emergency department setting for the MIMIC-IV dataset (Johnson et al., 2020). The detailed SOFA calculation is provided in Appendix Table 8. The baseline SOFA score is assumed to be zero unless additional information is available. The control class label is assigned to patients who meet neither of the two criteria, i.e., they do not have a suspicion of infection and do not experience a change in SOFA greater than 2 at any point during their ED or hospital stay.

The ASE label is assigned based on two sets of criteria: patients who have a suspicion of infection and those who have organ dysfunction. For suspicion of infection, patients must have at least four qualifying days of antibiotic use that overlap with the suspicion window surrounding a culture. The suspicion window starts 48 h before the culture order and ends 48 h after. For organ dysfunction, at least one of the following criteria must be met:

- Initiation of mechanical ventilator.
- Initiation of vasopressor.
- Doubling serum creatinine OR eGFR dropped by 50%.
- Doubling bilirubin.
- 50% decline in Platelet count or ≤ 100 cells/μL.

**Figure 1:**
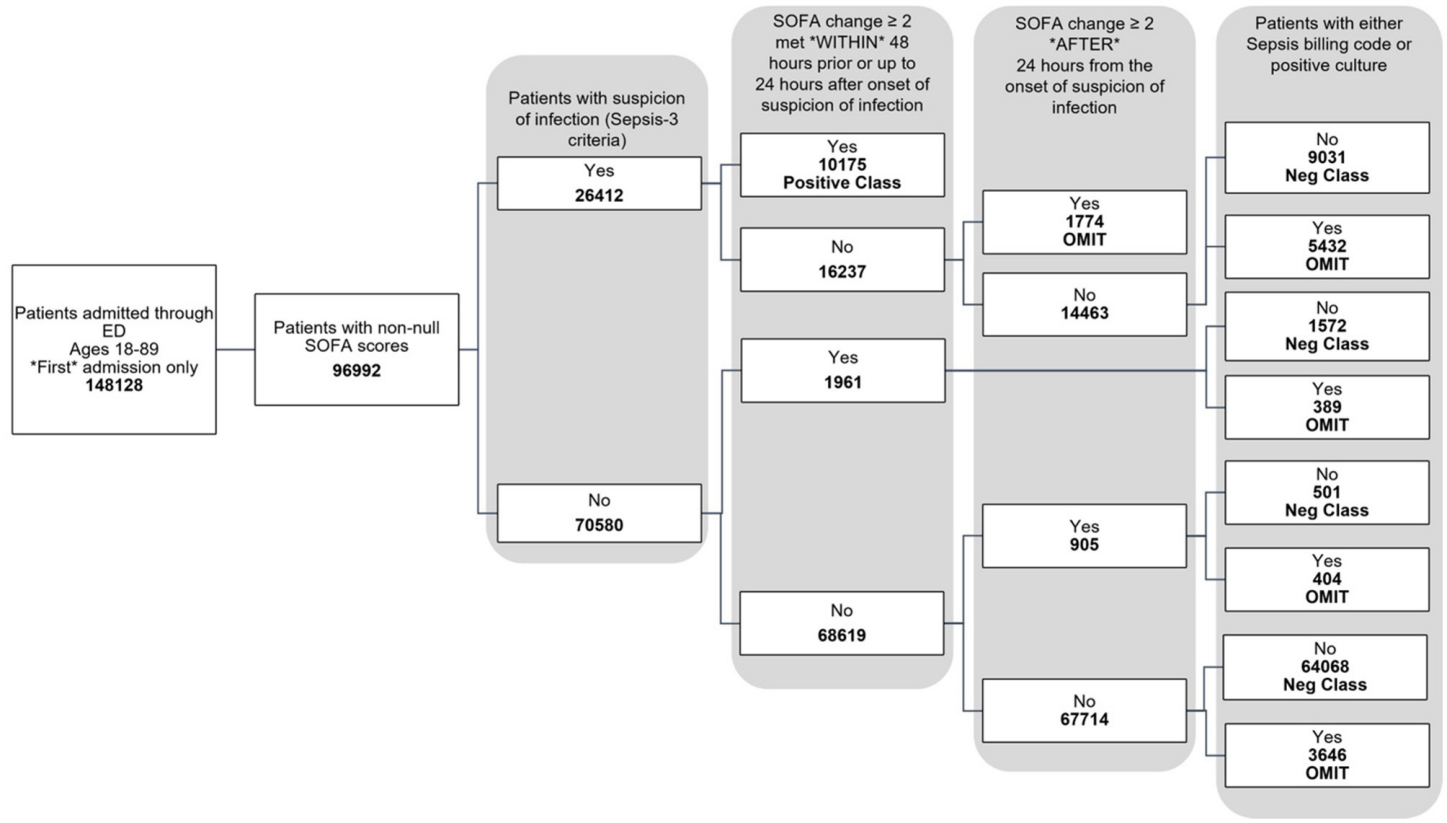
Consort diagram indicating the process of identification of patients with Sepsis-3 label.

**Figure 2:**
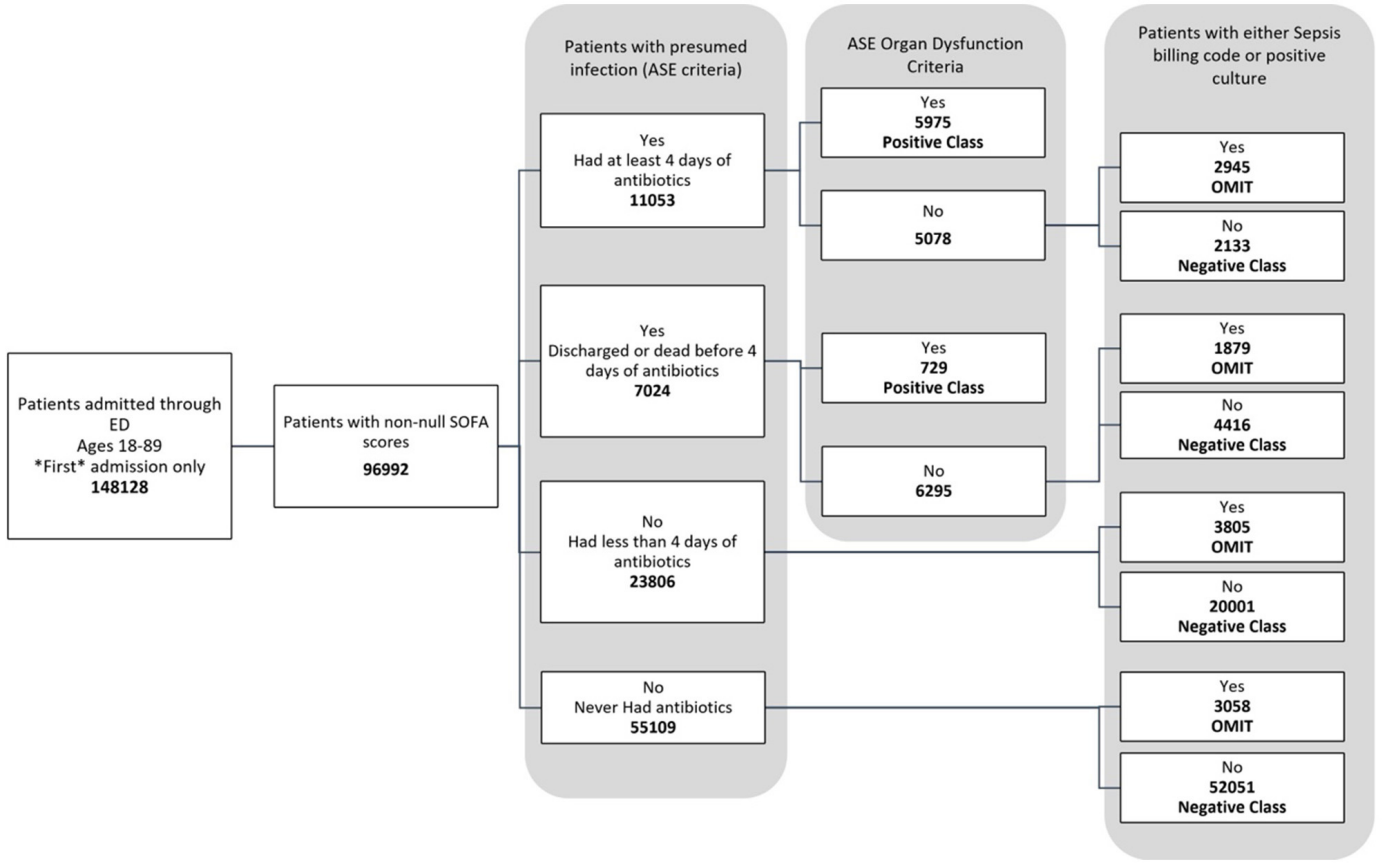
Consort diagram indicating the process of identification of patients with ASE (Adult Sepsis Event) label.

The data used for training is from the MIMIC-IV dataset (Johnson et al., 2020, 2023; Goldberger et al., 2000). Figure 1 shows the data flow for the Sepsis-3 label definition, while Figure 2 shows the consort diagram for the ASE label. We begin with 148,128 adult patients admitted through the emergency department, of which 96,992 patients have at least one of the following values:creatinine, bilirubin, platelet counts, or PaO2/FiO2, which are crucial for measuring organ dysfunction.

For the Sepsis-3 model, 10,175 patients are labeled as Sepsis-3 positive, and 75,712 patients are classified as controls. This cohort is split into training and testing datasets. We further restrict our analysis to cases with at least 50% non-null laboratory and vital values for training. As a result, the final training dataset consists of 32,823 patients (26,026 control and 6,801 Sepsis-3 labeled), and the test dataset contains 8,111 patients (6,598 control and 1,513 Sepsis-3 labeled).

A 10-fold cross-validation method is employed to train 10 models based on the training data to predict Sepsis-3 labels. The testing data is used solely for evaluation purposes and was not involved in model training or selection. Similarly, a second set of models is trained to predict the ASE label. The training set for these models includes 31,841 patients (27,939 control and 3,902 ASE labeled). The test dataset consists of 7,837 patients (6,916 controls and 921 ASE labeled). The features used in this model include routine blood tests such as CBC (Complete Blood Count), CMP (Comprehensive Metabolic Panel), and lipid panels, along with vital signs, age, and sex. We took into account the extent of missing data in the MIMIC-IV database, and features with significantly different levels of missingness between the control and disease populations were excluded to minimize potential bias in the model. The complete list of features used in the model is provided in Appendix Table 1. All blood measurements are collected within a specific patient encounter; no blood measurements are combined from different encounters on different dates. Clinical evidence supports that these values, measured at the time of admission to the emergency department, are strong indicators of sepsis, as outlined in the consensus paper on Sepsis definitions (Singer et al., 2016).

**Figure 3:**
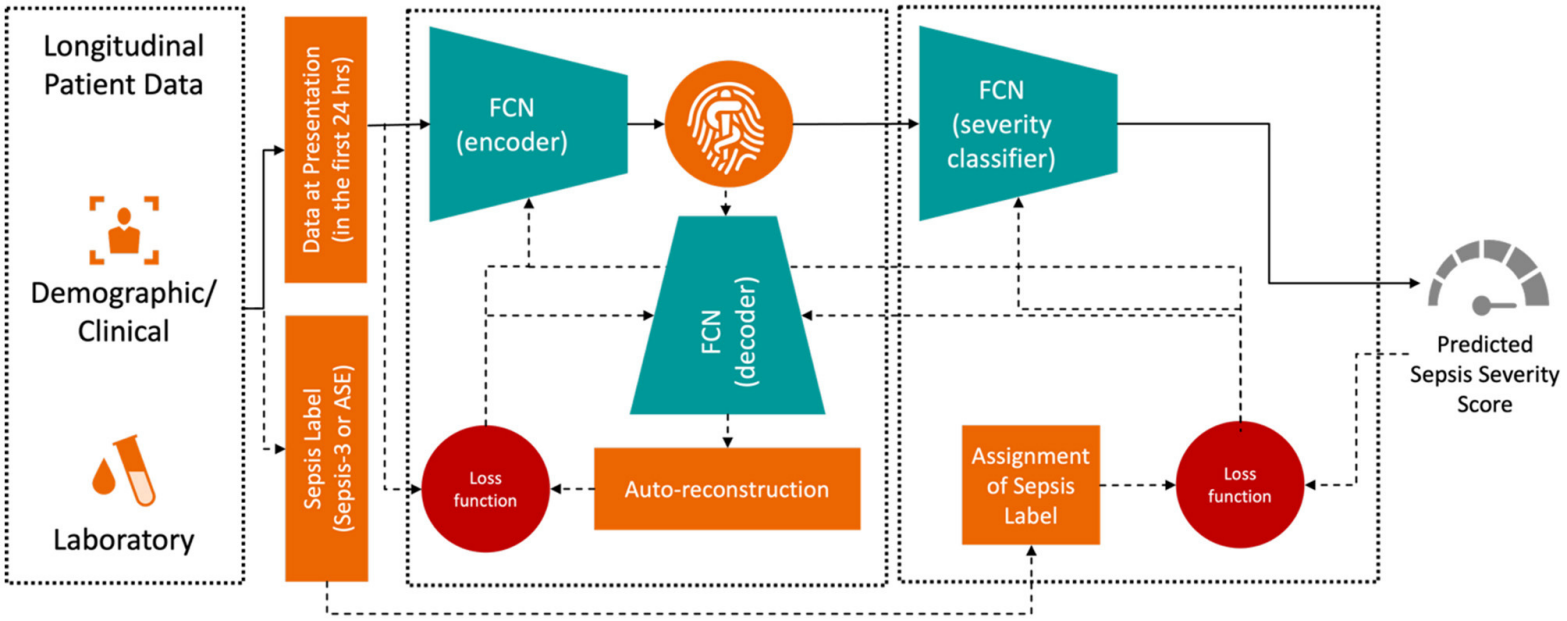
Model architecture for the deep profiler.

The training objective was to minimize the difference between the true and predicted sepsis likelihoods as output by the Deep Profiler. The network consists of a two-stage multi-layer perceptron, with its detailed architecture described in Singh et al. (2021). The input vector includes the median values for each laboratory and vital sign measurement within the first 24 h of admission. We train the Deep Profiler, shown in Figure 3, which learns a latent representation of the input vector and predicts a severity score for sepsis. The latent vector is learned using a variational autoencoder (VAE) framework, which generates a probability distribution for each latent attribute corresponding to an input instance. The encoder consists of three fully connected layers with channel sizes of 64, 32, and 32, each followed by a batch normalization layer and a leaky rectified linear activation (leaky ReLU) with a slope of 0.2. A decoder reconstructs the original vector from the latent representation to maintain the integrity of the input features. The decoder comprises three fully connected layers with channel sizes of 32, 32, and 64, each followed by a batch normalization layer and a rectified linear activation (ReLU). A classifier network is used to predict the likelihood of sepsis. The network consists of four fully connected layers. The first three layers each have 32 channels and are followed by a rectified linear activation (ReLU). The fourth fully connected layer maps the 32-channel feature to a single value (i.e., a calibrated softmax output; Niculescu-Mizil and Caruana, 2005) in the range of 0 to 1, where 0 indicates a very low likelihood of being diagnosed with sepsis and 1 indicates a very high likelihood of being diagnosed with sepsis in an emergency department. We performed a grid search with 10-fold internal cross-validation on the training data to identify the optimal hyperparameters. The best results were achieved with a batch size of 128, a learning rate of 3 × 10^−4^, and a dropout rate of 0.2. We also found that the model's performance was relatively stable, with minimal variation across the range of hyperparameters tested. The Adam optimizer was used for training.

## Results

The study was conducted on patients admitted through the emergency department, as captured in the MIMIC-IV database (Johnson et al., 2020, 2023; Goldberger et al., 2000). A total of 148,128 adults, aged 18 to 89 years, were included, of whom 96,992 had available laboratory data related to organ dysfunction. Only the first admission for each patient was considered. Among the 96,992 patients, 10,175 had a positive Sepsis-3 label, and 75,172 had a negative label, as shown in Figure 1. Additionally, 11,645 patients were assigned neither a positive nor negative label during training, as they could not be classified as Sepsis-3 positive based on the official criteria, but later had either a sepsis billing code (Appendix Table 4) or a positive culture. It was unclear whether these patients should be classified as negative for sepsis.

Figure 2 illustrates the flowchart for the ASE cohort. Of these, 6,704 patients were labeled as positive, while 78,601 patients were labeled as negative because they did not meet the criteria for organ dysfunction, were never coded for sepsis, and never had a positive culture sample. Another 11,687 patients were omitted from this definition as they had a sepsis billing code or a positive culture, even though they technically did not meet the criteria for an ASE positive label.

### Sepsis-3 and ASE labels concordance

Sepsis-3 and ASE use different criteria for presumed infection. According to ASE criteria, 11,053 cases (11.4%) are identified with presumed infections due to the administration of antibiotics for at least four consecutive days within 48 h of a culture analysis. 7,024 subjects (7.2%) either died or were discharged before completing a four-day antibiotic course and undergoing a culture test, thus qualifying as presumed infection under ASE criteria. Notably, both ASE and Sepsis-3 criteria align within these two categories. However, there is disagreement in some cases: Of the 23,806 individuals who received less than four days of antibiotics, 8,335 (35%) had a culture order within 48 h, making them positive for suspicion of infection according to Sepsis-3 criteria. This leads to a divergence from the ASE criteria. Finally, 55,109 subjects (56.6%) showed no suspicion of infection, a consistency across both definitions.

The ASE definition aligns with the Sepsis-3 negative label in 95% of cases. However, of the 10,175 Sepsis-3 positive subjects, only 5,308 (52.2%) are also positive by ASE criteria, while 4,867 (47.8%) show disagreement. To explore this discrepancy, we conduct a large-scale statistical EHR phenotyping of the subjects with disagreement in the positive label, utilizing tools provided by pyPheWAS (Kerley et al., 2022). pyPheWAS employs PheCode (Wu et al., 2019), an aggregation of ICD-9 and ICD-10 billing codes, to identify clinical phenotypes.

**Figure 4:**
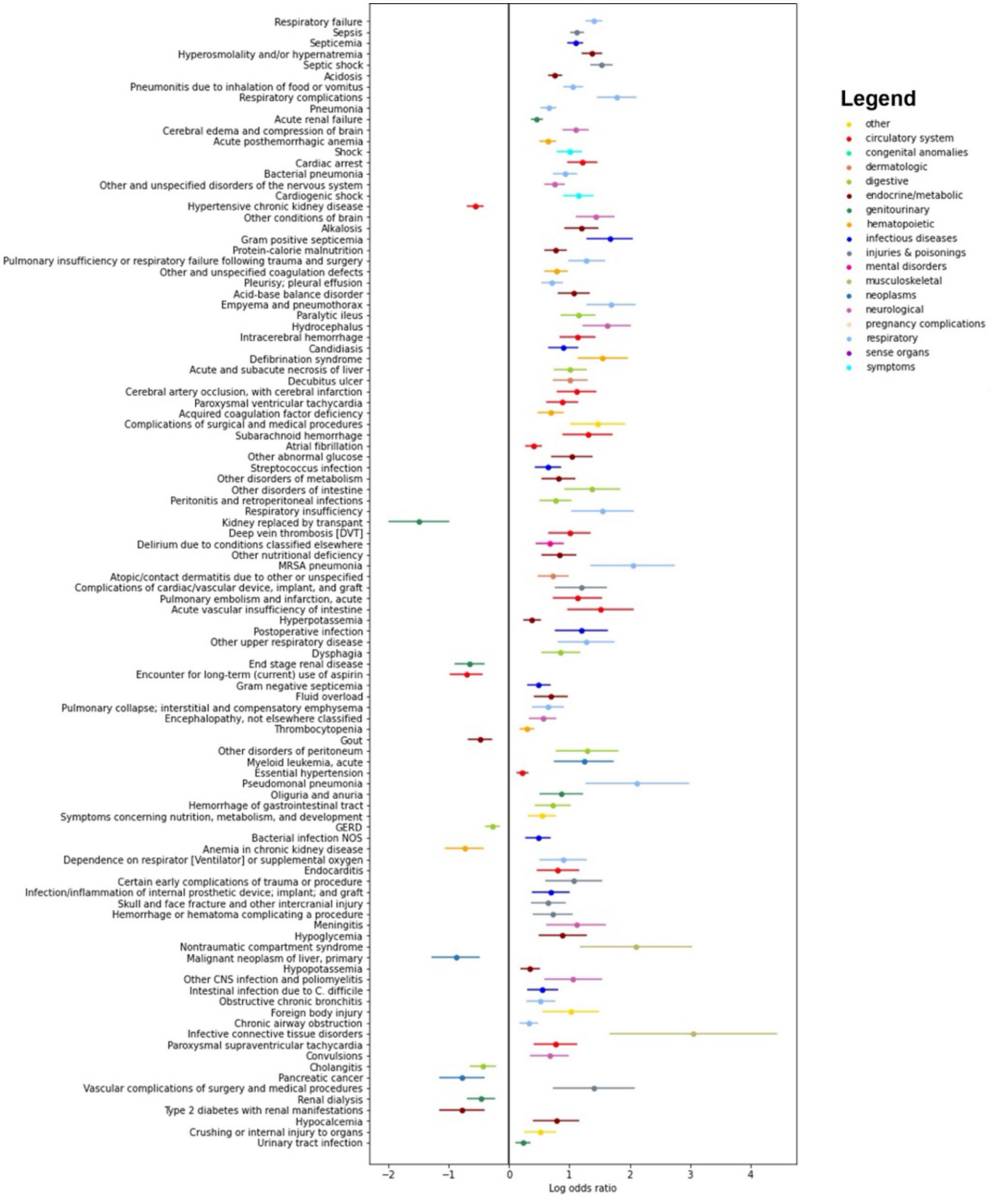
Results of the pyPheWAS analysis between the cohort of patients who are positive both with Sepsis-3 and ASE **(right hand side)** and the cohort where Sepsis-3 is positive and ASE is negative **(left hand side)**. The analysis shows which conditions are more likely in each of the two cohorts. We see that chronic diseases which can result in organ dysfunction are more likely to be present in patients who have a positive Sepsis-3 label but negative with ASE.

Figure 4 presents log odds plots for clinical phenotypes more likely to be found in each group. On the right side, we observe conditions more common in patients with both Sepsis-3 and ASE labels positive, such as infections, acute organ failures, sepsis, and septicemia. On the left side, conditions more common in patients with a Sepsis-3 positive label but an ASE negative label include underlying kidney, liver, and coagulation disorders, as well as hemorrhages. These conditions could result in a higher SOFA score, leading to misidentification as sepsis due to the higher SOFA score and the weaker presumption of infection criteria in the Sepsis-3 definition.

### Performance of the models for early risk prediction

**Figure 5:**
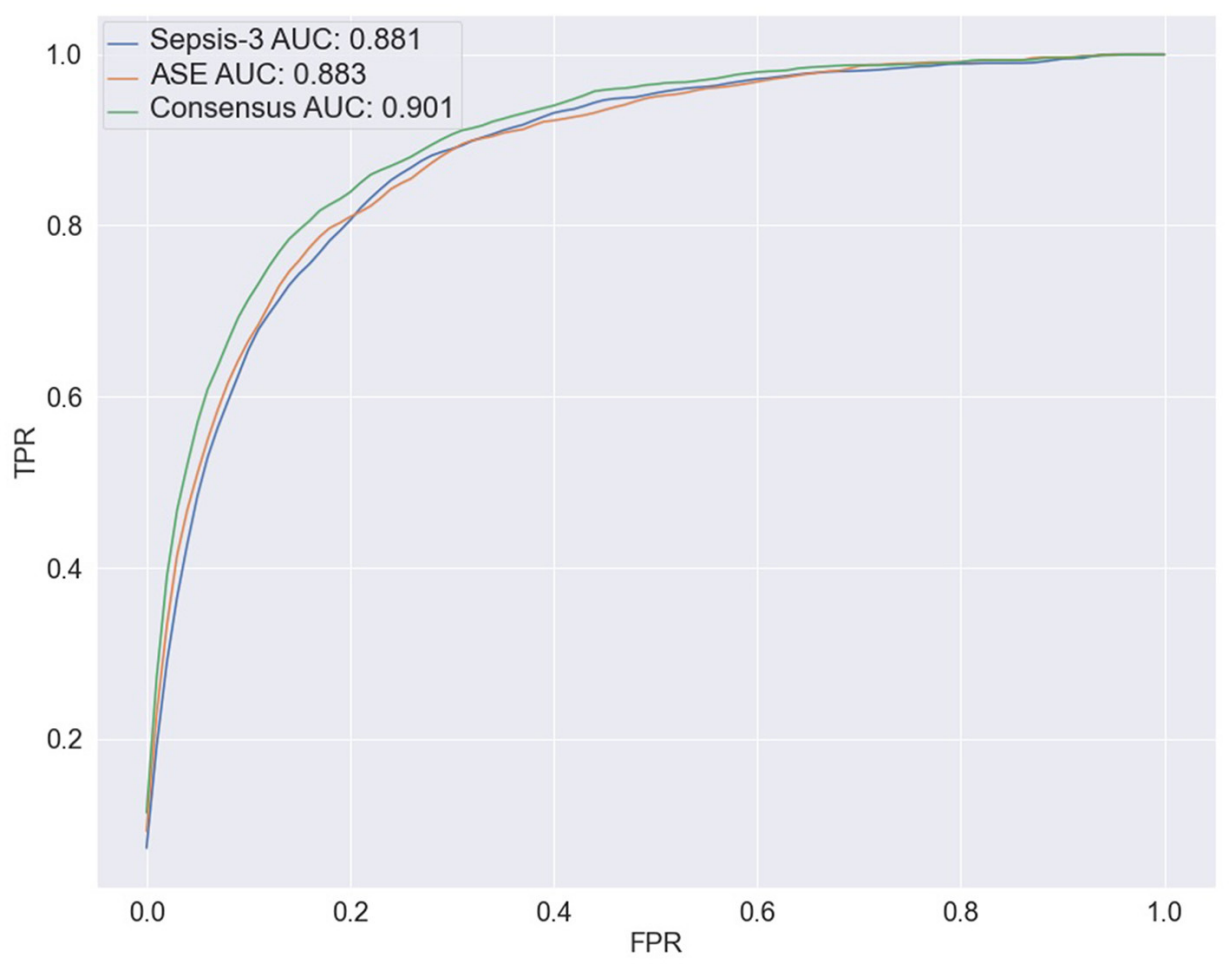
The ROC curves for the three ensemble models are shown here. The blue line represents the results from combining the output of 10 models trained with the Sepsis-3 label, which has an AUC of 0.881. The orange line is used to represent the ASE ensemble model, with an AUC of 0.883. The Consensus model is represented by the green line with an increased AUC of 0.900.

To predict the risk of sepsis, we trained two separate models: one based on Sepsis-3 criteria and the other on ASE criteria. The features included in the models comprised laboratory measurements and vital signs (see Appendix Table 1 for a complete list). We employed a deep neural network (DNN) known as the deep profiler (Singh et al., 2021) to train the models. Each model demonstrated similar performance individually, with both achieving an AUC of 0.88. When specificity was set to 80%, the Sepsis-3 model had a sensitivity of 80.5%, while the ASE model had a sensitivity of 80.9%. Appendix Table 2 presents the performance metrics along with confidence intervals. Although the performance differences between the two models were not statistically significant, we observed a statistically significant (Appendix Table 2b) improvement when the models were combined, as shown in Figure 5. The ground truth for the consensus model is derived from the combined Sepsis-3 and ASE labels. The AUC increased to 0.9, and sensitivity rose to 83.7%.

**Figure 6:**
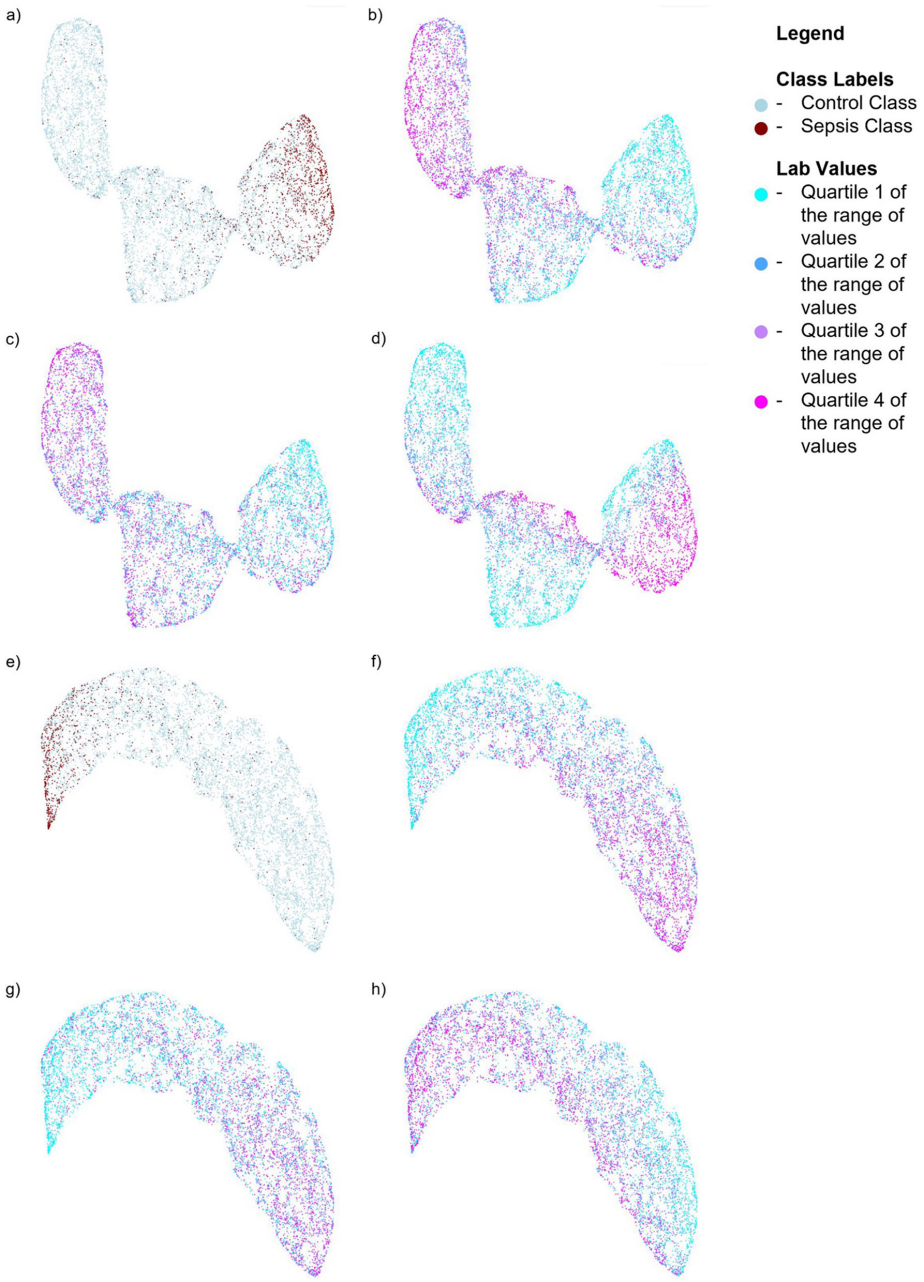
UMAPs for the Sepsis-3 and ASE models showing the distribution of class labels and the top three labs contributing to the respective models. For the model trained on Sepsis-3 labels: **(a)** Shows the distribution of the class labels **(b)** Shows the distribution of calcium lab values **(c)** Shows the distribution of platelet counts. **(d)** Shows the distribution of creatinine lab values. For the model trained on ASE labels: **(e)** Shows the distribution of ASE class labels. **(f)** Shows the distribution of calcium lab values. **(g)** Shows the distribution of bicarbonate values. **(h)** Shows the distribution of glucose lab values.

We also performed SHAP analysis (Lundberg and Lee, 2017) to determine the contribution of each feature to the models (see Appendix Figures 1, 2). In the Sepsis-3 model, the top three most important features were Calcium, Creatinine, and Platelet values—two of which are used in the calculation of SOFA scores. Low calcium levels are commonly observed in patients with sepsis (Zivin et al., 2001). The three most important features for the ASE model are calcium, glucose, and bicarbonate values. To visualize the distribution of the classes in a low-dimensional space, we computed UMAPs (McInnes et al., 2018), which help to see how the significant lab values are distributed in relation to the class labels. Figure 6 illustrates the distribution of Sepsis-3 class and control labels in this reduced space. The region with a high concentration of Sepsis-3 labels is associated with lower calcium levels, elevated creatinine, and reduced platelet count. Interestingly, we observe that regions with hypocalcemia, high creatinine, and low platelet counts overlap with both Sepsis-3 and control regions. This overlap indicates that there could be various reasons for changes in these lab values. The value of the model lies in correctly identifying when these changes are due to sepsis rather than other confounding clinical factors. Similarly, Figure 6 shows that the region with a high concentration of ASE labels corresponds to areas with lower calcium and bicarbonate levels and elevated glucose.

### Performance by cohort

Sepsis is a condition characterized by a dysregulated immune response to severe infection, leading to sequential organ failure. Identifying sepsis in the emergency department is particularly challenging due to the diverse presentations of patients and the lack of detailed patient history. A high SOFA score, which indicates organ dysfunction, can result from either acute sepsis or pre-existing chronic conditions such as chronic kidney disease or liver disease, making it difficult to distinguish between the two in real-time or retrospective analyses. This ambiguity complicates the evaluation of sepsis prediction models. On the other hand, certain cohorts are less prone to misclassification. For instance, patients with confirmed infections (validated by infection-related billing codes) or those with a confirmed Sepsis billing code are more likely to have SOFA score increases that are attributable to sepsis, rather than unrelated chronic conditions. This makes them more suitable for robust model evaluation. To assess model performance across these diverse subgroups, we analyze performance metrics based on EHR phenotypes as follows:

- Cohorts with confounding conditions: these cohorts encompass diseases capable of causing organ failure, leading to elevated SOFA scores. There are three specific confounding cohorts:

- Underlying or chronic kidney disease, which can impact creatinine or BUN labs (detailed in Appendix Table 5).
- Chronic or underlying liver disease, affecting albumin, bilirubin, PTT, or INR labs (detailed in Appendix Table 7).
- Hemorrhage or underlying coagulation conditions, which may influence platelet counts, INR, or PTT (detailed in Appendix Table 6).

- Cohort with Infection Billing Codes (detailed in Appendix 8).
- Cohort with All Test Subjects, excluding those with confounding conditions.
- Cohort with Sepsis Billing Codes Only (detailed in Appendix Table 4).

In Table 1, cohorts with confounding conditions show notably higher sensitivity compared to the overall sensitivity. For this comparison, we only included patients with Sepsis-3 and ASE labels in agreement to eliminate potential bias from label errors and provide a fair evaluation of how cohort characteristics and individual labels impact the model. However, this results in a higher false positive rate. Additionally, patients with underlying kidney conditions have the lowest Positive Predictive Value (PPV) at 22.9% for the consensus model. The cohort with hemorrhage or coagulation conditions demonstrates the highest sensitivity, reaching 90.3% for the consensus model. Within the cohort of patients with infection billing codes, overall sensitivity remains high at 87.9%, with a PPV of 77%. However, the Negative Predictive Value (NPV) drops to 85.1%, as the model occasionally misclassifies infected patients as having sepsis. The cohort excluding confounding conditions shows higher specificity compared to the entire test dataset. Notably, sepsis billing codes are known for their low sensitivity but high PPV and specificity (Henry et al., 2019). The models perform the best for the cohort with sepsis billing codes, as shown in Table1, with the consensus model achieving the highest sensitivity of 93.1%.

**Table 1:** Comparison of performance among different sub-cohorts.

**Training Label**	**Entire Cohort**	**Without confounders**		**With confounders**		
		**Infection billing code only**	**Sepsis billing code only**	**Chronic or underlying kidney disease**	**Hemorrhage or chronic coagulapathy**	**Chronic or underlying liver disease**
**Sepsis 3**	AUC = 0.880					
	SE = 0.820	SE = 0.863	SE = 0.923	SE = 0.896	SE = 0.896	SE = 0.839
	SP = 0.802	SP = 0.733		SP = 0.460	SP = 0.531	SP = 0.704
	PPV = 0.357	PPV = 0.772		PPV = 0.223	PPV = 0.452	PPV = 0.351
	NPV = 0.971	NPV = 0.836		NPV = 0.963	NPV = 0.922	NPV = 0.958
**ASE**	AUC = 0.881					
	SE = 0.826	SE = 0.869	SE = 0.923	SE = 0.842	SE = 0.899	SE = 0.871
	SP = 0.824	SP = 0.768		SP = 0.603	SP = 0.569	SP = 0.747
	PPV = 0.387	PPV = 0.797		PPV = 0.268	PPV = 0.473	PPV = 0.397
	NPV = 0.972	NPV = 0.848		NPV = 0.957	NPV = 0.929	NPV = 0.968
**Sepsis 3 ASE Consensus**	AUC = 0.901					
	SE = 0.837	SE = 0.879	SE = 0.931	SE = 0.898	SE = 0.903	SE = 0.839
	SP = 0.800	SP = 0.725		SP = 0.479	SP = 0.528	SP = 0.704
	PPV = 0.359	PPV = 0.770		PPV = 0.229	PPV = 0.452	PPV = 0.351
	NPV = 0.973	NPV = 0.851		NPV = 0.964	NPV = 0.927	NPV = 0.958

## Discussion

Sepsis causes organ failure due to a dysregulated immune response to infection and is typically assessed by evaluating the function of multiple organs based on Sepsis-3 or ASE criteria. Our investigation focused on the robustness of various sepsis definitions and the efficacy of ensemble classifiers in identifying patients with severe illness. In the emergency department, distinguishing between acute sepsis and pre-existing chronic organ dysfunction is particularly challenging without access to patient history. Comorbidities such as chronic kidney disease, liver disease, or coagulation disorders complicate retrospective labeling and model evaluation. Our comprehensive analysis, encompassing diverse patient cohorts, highlights the difficulty of achieving consistent performance in a heterogeneous sepsis population characterized by numerous confounding conditions. The variable performance across different cohorts emphasizes the need for tailored approaches in sepsis diagnosis, particularly when managing patients with complicating comorbidities.

We note that the models presented in this paper are based on the median of the laboratory values available in the first 24 h of patient admission. In practice, emergency departments may have already flagged some patients as being suspected of having sepsis, as evidenced by the placement of culture orders for a subset of these patients. However, we chose to include all laboratory values from the first 24 h for all patients to simplify the comparison of model performance across different labels and comorbidity cohorts. A causal evaluation of model performance, based on the timing and presence or absence of culture orders, is provided in the Appendix Table 3 for additional clarity. We show that the model is able to identify 88% of the cases that receive a delayed culture order within 24 h. This suggests that, regardless of the training label used, the model's output is informative for cases where care providers might not have suspected an infection. Comparing our model to existing sepsis models is inherently complex due to differences in patient populations, clinical settings, and predicted outcomes. Most existing models focus on hospital-onset sepsis in ICU settings, which differs significantly from our focus on community-onset sepsis in emergency departments. Retrospective labeling inconsistencies further complicate these comparisons. However, when compared to Liu et al. (2025), who used Sepsis-3 criteria and the MIMIC-IV dataset in emergency department settings, our model achieves a higher AUC (0.9 vs. 0.83).

Deploying predictive models in emergency settings also raises significant ethical considerations. Performance variability across sub-cohorts, particularly for patients with confounding conditions, may lead to disparities in care. While SHAP analysis offers valuable insights into feature importance, real-time interpretability is crucial for building clinician trust and ensuring the model is used effectively. Furthermore, false positives can contribute to alert fatigue, potentially leading to unnecessary interventions and misallocation of resources.

The insights from this paper contribute to the ongoing discussion on sepsis management and emphasize the need for individualized assessments, especially for patients with specific underlying health conditions. In future work, we will focus on prospective validation and implementing post-deployment strategies, including real-time monitoring, clinician training, and optimizing alert thresholds to balance sensitivity and specificity. A key area of our future research will be the development of personalized machine learning models tailored to each patient's unique comorbidities. The concepts and information presented in this paper/presentation are based on research results that are not commercially available. Future commercial availability cannot be guaranteed.

## Data Availability

Publicly available datasets were analyzed in this study. This data can be found here: https://physionet.org/content/mimiciv/2.2/.
