# Supplementary material for: Evaluating the impact of common clinical confounders on performance of deep-learning-based sepsis risk assessment
Source: Front Artif Intell. 2025 Jul 15;8:1452471. doi: 10.3389/frai.2025.1452471 (PMC12305701; doi:10.3389/frai.2025.1452471)

**Appendix Table 1.** Features of the model

| Type | Feature |
| --- | --- |
| Demographics | Age, Gender |
| Vital signs | Temperature, Respiration, Systolic and Diastolic BP, O2 Saturation, MAP, Heartrate |
| Laboratory measurements | BUN, Calcium, Chloride, Creatinine, CRP, Ferritin, Glucose, Bicarbonate, Hematocrit, Hemoglobin, INR, Magnesium, Phosphate, Platelets, Potassium, PTT, Sodium |

**Appendix Table 2(a)** Performance metrics along with the 95^th^ percentile Interval for the models trained on Sepsis-3, ASE and consensus label, given specificity of 0.8.

| Model | Performance metrics |
| --- | --- |
| Model Trained using Sepsis 3 Label | AUC: 0.881 [0.872,0.889]  SE: 0.805 [0.783,0.828]  **SP: 0.800** ,  PPV: 0.480 [0.465, 0.494]  NPV: 0.947 [ 0.941, 0.953] |
| Model Trained using ASE | AUC: 0.883 [0.873, 0.893]  SE: 0.809 [0.785, 0.831]  **SP: 0.800**  PPV: 0.349 [0.334, 0.365]  NPV: 0.971 [0.966, 0.973] |
| Model Inference using Sepsis 3 and ASE consensus | AUC: 0.9000 [0.891, 0.909]  SE: 0.837 [0.814, 0.860]  **SP: 0.800**  PPV: 0.359 [0.343, 0.374]  NPV: 0.973 [0.969, 0.977] |

**Table 2(b)** Results of a one-sided t-test to compute the significance of the improvement of the consensus model over the Sepsis-3 or the ASE model. ** indicates statistically significant improvement (p<0.0001). All metrics show significant improvement except PPV for Sepsis-3 model. PPV for the Sepsis-3 model is better than the consensus model.

|  | p-value | | | |
| --- | --- | --- | --- | --- |
|  | AUC | Sensitivity | PPV | NPV |
| Consensus vs Sepsis-3 | $\boldsymbol{**p=7.7}\boldsymbol{e-36}$ | $\boldsymbol{**p=1.8}\boldsymbol{e-18}$ | $p=1$ | $\boldsymbol{p=2.99}\boldsymbol{e-89}$ |
| Consensus vs ASE | $\boldsymbol{**p=1.6}\boldsymbol{e-27}$ | $\boldsymbol{**p=1.1}\boldsymbol{e-15}$ | $\boldsymbol{**p=4.2}\boldsymbol{e-06}$ | $\boldsymbol{**p=6.3}\boldsymbol{e-7}$ |

**Appendix Table 3.** Performance for patients with a delayed culture order

604 patients get a culture order after the first 24 hours, of which 41% have Sepsis. For these 604 patients, we compute the performance of the model based on the labs in the first 24 hours, i.e., before the hospital suspects Sepsis or orders culture. To compare to the hospital performance, we choose an operating point such that it has the same precision as the Hospital decision, i.e. 41%. At this operating point, the model can identify 88% of people with sepsis within the first 24 hours.

(a)

| Model prediction | Ground truth concordant Sepsis label | |
| --- | --- | --- |
|  | False | True |
| False | 283 | 17 |
| True | 178 | 226 |

(b)

| Model performance in identifying patients with delayed culture order | AUC: 0.84  SE: 0.88  SP: 0.61  PPV: 0.41  NPV: 0.94 |
| --- | --- |

**Appendix Table 4.** Sepsis codes

| **SEPSIS** | **ICD-9** | **ICD-10** |
| --- | --- | --- |
| Sepsis | 995.91/995.92 | A26.7/A41.9/A42.7 |
| Septicemia | 038/038.3/038.8/038.9/054.5/449 | A41.4/A41.8/A41.9/A42.7/B00.7 |
| Septic shock | 785.52 | nan |
| Gram positive septicemia | 022.3/038.1/038.10/038.11/038.12/038.19/038.2 | A22.7/A40.3/A41.0/A41.1/A41.2 |
| Gram negative septicemia | 003.1/020.2/036.2/038.0/038.4/038.40/038.41/038.42/038.43/038.44/038.49 | A02.1/A20.7/A39.2/A39.3/A39.4/A40/A40.0/A40.1/A40.2/A40.8/A40.9/A41.3/A41.5 |

**Appendix Table 5.** Billing codes for underlying kidney disease

| **Underling Kidney Disease** | **ICD-9** | **ICD-10** |
| --- | --- | --- |
| Hypertensive chronic kidney disease | 403/403.0/403.00/403.01/403.1/403.10/403.11/403.9/403.90/403.91 | I12/I12.0/I12.9 |
| Kidney replaced by transplant | 996.81/V42.0 | Z94.0 |
| End stage renal disease | 585.5/585.6 | nan |
| Renal dialysis | 792.5/996.73/E870.2/E871.2/E872.2/E874.2/E879.1/V45.1/V45.11/V45.12/V56/V56.0/V56.1/V56.2/V56.31/V56.32/V56.8 | Y60.2/Y61.2/Y62.0/Y84.1/Z49.1/Z49.2/Z99.2 |
| Chronic Kidney Disease, Stage III | 585.3 | nan |
| Chronic renal failure [CKD] | 585/585.0/585.9 | N18/N18.0/N18.9 |
| Hypertensive heart and/or renal disease | 404/404.0/404.00/404.01/404.02/404.03/404.1/404.10/404.11/404.12/404.13/404.9/404.90/404.91/404.92/404.93 | I13/I13.0/I13.1/I13.2/I13.9 |
| Chronic Kidney Disease, Stage IV | 585.4 | nan |
| Cystic kidney disease | 753.1/753.10/753.11/753.12/753.13/753.14/753.15/753.16/753.17/753.19 | Q61/Q61.0/Q61.1/Q61.2/Q61.3/Q61.4/Q61.5/Q61.8/Q61.9 |

**Appendix Table 6.** Billing codes for hemorrhage/coagulation conditions

| **Hemorrhage/Coagulation disorder** | **ICD-9** | **ICD-10** |
| --- | --- | --- |
| Acute posthemorrhagic anemia | 285.1 | D62 |
| Acquired coagulation factor deficiency | 286.7 | D68.4 |
| Myeloid leukemia, acute | 205.0/205.00/205.01/205.02 | C92.0/C92.4/C92.5/D46.3 |
| Hemorrhage of gastrointestinal tract | 578.9 | K92.2 |
| Hemorrhage or hematoma complicating a procedure | 958.2/998.1/998.11/998.12/998.13 | T81.0 |
| Esophageal bleeding (varices/hemorrhage) | 456.0/456.1/456.2/456.20/456.21/530.82 | I85/I85.0/I85.9/I98.2/K22.8 |
| Secondary malignancy of lymph nodes | 196/196.0/196.1/196.2/196.3/196.5/196.6/196.8/196.9/209.71 | C77/C77.0/C77.1/C77.2/C77.3/C77.4/C77.5/C77.8/C77.9 |
| Splenomegaly | 789.2 | R16/R16.1/R16.2 |

**Appendix Table 7.** Billing codes for underlying liver disease

| **Underlying Liver condition** | **ICD-9** | **ICD-10** |
| --- | --- | --- |
| Malignant neoplasm of liver, primary | 155.0/V10.07 | C22.0/C22.2/C22.3/C22.4/C22.7 |
| Cirrhosis of liver without mention of alcohol | 571.5 | K74.0/K74.6 |
| Secondary malignant neoplasm of liver | 197.7/209.72 | C78.7 |
| Malignant neoplasm of gallbladder and extrahepatic bile ducts | 156/156.0/156.1/156.2/156.8/156.9 | C23/C24/C24.0/C24.1/C24.8/C24.9 |

**Appendix Table 8.** Billing codes for infection

| **INFECTION** | **ICD-9** | **ICD-10** |
| --- | --- | --- |
| Pneumonia | 130.4/483/483.8/484/484.8/486/V12.61 | B58.3/J10.0/J11.0/J16.8/J17.2/J18/J18.8/J18.9 |
| Bacterial pneumonia | 003.22/020.3/020.4/020.5/021.2/022.1/031.0/039.1/073.0/083.0/482/482.0/482.2/482.3/482.30/482.31/482.32/482.39/482.4/482.40/482.42/482.49/482.8/482.81/482.82/482.83/482.84/482.89/482.9/483.0/483.1/484.3/484.5 | A20.2/A21.2/A22.1/A31.0/A42.0/A43.0/A48.1/A78/B96.0/B96.1/J14/J15.0/J15.2/J15.3/J15.4/J15.5/J15.6/J15.7/J15.8/J15.9/J16.0 |
| Candidiasis | 112/112.0/112.1/112.2/112.5/112.8/112.82/112.84/112.85/112.89/112.9 | B20.4/B37/B37.0/B37.3/B37.4/B37.7/B37.8/B37.9 |
| Streptococcus infection | 034/034.1/041.0/041.00/041.01/041.02/041.03/041.04/041.05/041.09 | A38/A40.1/A40.2/A49.1/B95.0/B95.1/B95.2/B95.3/B95.4/B95.5/J03.0/J20.2/M00.2 |
| MRSA pneumonia | 482.41 | nan |
| Postoperative infection | 519.01/530.86/536.41/998.5/998.51/998.59/999.3/999.39 | N98.0/T81.4/T88.0 |
| Pseudomonal pneumonia | 482.1 | J15.1 |
| Bacterial infection NOS | 020/020.0/020.8/020.9/021/021.8/021.9/022/022.8/022.9/023/023.0/023.1/023.2/023.3/023.8/023.9/024/025/026/026.0/026.9/027/027.0/027.1/027.2/027.8/027.9/032/032.8/032.89/032.9/033/033.0/033.1/033.8/033.9/036/036.3/036.8/036.81/036.89/036.9/037/039/039.2/039.3/039.4/039.8/039.9/040/040.0/040.1/040.2/040.3/040.4/040.42/040.8/040.81/040.82/040.89/041/041.2/041.3/041.5/041.6/041.7/041.8/041.81/041.82/041.83/041.84/041.85/041.89/041.9/795.31 | A15.9/A20/A20.0/A20.3/A20.8/A20.9/A21/A21.7/A21.8/A21.9/A22/A22.8/A22.9/A23/A23.0/A23.1/A23.2/A23.3/A23.8/A23.9/A24/A24.0/A24.1/A24.2/A24.3/A24.4/A25/A25.0/A25.9/A26/A26.0/A26.7/A26.8/A26.9/A28.0/A28.2/A28.8/A28.9/A32/A32.0/A32.1/A32.7/A32.8/A32.9/A35/A36/A36.8/A36.9/A37/A37.0/A37.1/A37.9/A39/A39.1/A39.8/A39.9/A42/A42.1/A42.2/A42.8/A42.9/A43/A43.8/A43.9/A48.0/A48.2/A48.3/A48.4/A48.8/A49/A49.2/A49.3/A49.8/A49.9/B47/B47.1/B47.9/B96.3/B96.4/B96.5/B96.6/B96.7/B96.8/J20.0/J20.1/M00.1 |
| Infection/inflammation of internal prosthetic device; implant; and graft | 996.6/996.60/996.61/996.62/996.63/996.64/996.65/996.66/996.67/996.68/996.69/999.31 | T82.6/T83.5/T83.6/T84.6/T84.7/T85.7 |
| Other CNS infection and poliomyelitis | 045/045.0/045.00/045.01/045.02/045.03/045.1/045.10/045.11/045.12/045.13/045.2/045.20/045.21/045.22/045.23/045.9/045.90/045.91/045.92/045.93/046/046.0/046.3/046.7/046.71/046.72/046.79/046.8/046.9/048/048.0/049/138/138.0/324/324.0/324.1/324.9/326/V12.02/V12.42 | A80/A80.0/A80.1/A80.2/A80.3/A80.4/A80.9/A81/A81.2/A81.8/A81.9/A88.0/B91/G06/G06.0/G06.1/G06.2/G07/G09/I67.3 |
| Intestinal infection due to C. difficile | 8.45 | A04.7 |
| Infective connective tissue disorders | 567.31/710.5/728.0/728.86 | M60.0 |
| Pneumococcal pneumonia | 481/481.0 | J13/J18.1 |
| Bronchopneumonia and lung abscess | 485/485.0/513/513.0/513.1/517.1 | J18.0/J85/J85.0/J85.1/J85.2/J85.3 |
| Mycoses | 117/117.0/117.1/117.2/117.4/117.5/117.6/117.7/117.8/117.9/118 | B42/B42.0/B42.1/B42.7/B42.8/B42.9/B43.0/B43.1/B43.2/B43.8/B43.9/B45/B45.0/B45.2/B45.3/B45.7/B45.8/B45.9/B46/B46.0/B46.1/B46.2/B46.3/B46.4/B46.5/B46.8/B46.9/B47.0/B48.1/B48.2/B48.3/B48.7/B48.8/B49/G02.1/J17.2 |
| Aspergillosis | 117.3/518.6 | B44/B44.0/B44.1/B44.2/B44.7/B44.8/B44.9/B48.4 |
| Acute upper respiratory infections of multiple or unspecified sites | 032.0/032.1/032.2/032.3/034.0/460/464.3/464.30/464.31/464.5/464.50/464.51/465/465.0/465.8/465.9 | A36.0/A36.1/A36.2/J00/J02.0/J03.0/J05.1/J06/J06.0/J06.8/J06.9 |
| Viral Enteritis | 008.6/008.61/008.62/008.63/008.64/008.65/008.66/008.67/008.69/008.8 | A08/A08.0/A08.1/A08.2/A08.3/A08.4/A08.5 |
| Infection with drug-resistant microorganisms | V09/V09.0/V09.1/V09.2/V09.3/V09.4/V09.5/V09.50/V09.51/V09.6/V09.7/V09.70/V09.71/V09.8/V09.80/V09.81/V09.9/V09.90/V09.91 | nan |
| Pneumonia due to fungus (mycoses) | 112.4/114.0/114.4/114.5/136.3/484.6/484.7 | B20.6/B37.1/B38.0/B38.1/B38.2/B44.0/B59/J17 |
| Methicillin sensitive Staphylococcus aureus | 41.11 | nan |
| Staphylococcus infections | 041.1/041.10/041.19 | A49.0/B95.6/B95.7/B95.8/M00.0 |
| Herpes simplex | 054/054.0/054.1/054.10/054.11/054.12/054.13/054.19/054.2/054.6/054.7/054.71/054.73/054.74/054.79/054.8/054.9/058/058.1/058.10/058.11/058.12/058.8/058.81/058.82/058.89 | A60/A60.0/A60.1/A60.9/B00/B00.0/B00.1/B00.2/B00.8/B00.9/B08.2 |
| Candidiasis of skin and nails | 112.3 | B37.2 |
| Methicillin resistant Staphylococcus aureus | 041.12/V12.04 | nan |
| Influenza | 487/487.0/487.1/487.8/488/488.0/488.01/488.02/488.09/488.1/488.11/488.12/488.19/488.81/488.82/488.89 | J10.0/J10.1/J10.8/J11.0/J11.1/J11.8 |
| Viral infection | 050/050.0/050.1/050.2/050.9/051/051.0/051.01/051.02/051.1/051.2/051.9/055/055.7/055.79/055.8/055.9/056/056.0/056.00/056.09/056.7/056.79/056.8/056.9/057/057.0/057.8/057.9/059/059.0/059.00/059.01/059.09/059.1/059.10/059.11/059.12/059.19/059.2/059.20/059.21/059.22/059.8/059.9/060/060.0/060.1/060.9/061/065/065.0/065.1/065.2/065.3/065.4/065.8/065.9/066/066.0/066.1/066.3/066.4/066.40/066.41/066.42/066.49/066.8/066.9/071/072/072.0/072.3/072.7/072.71/072.72/072.79/072.8/072.9/073/073.7/073.8/073.9/074/074.0/074.1/074.3/074.8/078/078.0/078.2/078.3/078.4/078.5/078.6/078.7/078.8/078.81/078.82/078.88/078.89/079/079.0/079.1/079.2/079.3/079.5/079.50/079.51/079.52/079.59/079.6/079.8/079.81/079.82/079.83/079.88/079.89/079.9/079.98/079.99/999.0 | A28.1/A70/A74/A74.8/A74.9/A82/A82.0/A82.1/A82.9/A88.1/A90/A91/A92/A92.0/A92.1/A92.3/A92.4/A92.8/A92.9/A93/A93.0/A93.1/A93.2/A93.8/A94/A95/A95.0/A95.1/A95.9/A96/A96.0/A96.1/A96.2/A96.8/A96.9/A98/A98.0/A98.1/A98.2/A98.3/A98.4/A98.5/A98.8/A99/B00.8/B03/B04/B05/B05.1/B05.4/B05.8/B05.9/B06/B06.0/B06.8/B06.9/B08.0/B08.1/B08.3/B08.4/B08.5/B08.8/B09/B20.2/B23.0/B25/B25.2/B25.8/B25.9/B26/B26.0/B26.3/B26.8/B26.9/B33.0/B33.1/B33.3/B33.8/B34/B34.0/B34.1/B34.2/B34.3/B34.4/B34.8/B34.9/B97.0/B97.1/B97.3/B97.4/B97.5/B97.6/B97.7/B97.8/H81.2/J20.3/J20.4/J20.5/J20.6/J20.7/K67.0/L44.4 |
| Other infectious and parasitic diseases | 080/081/081.0/081.1/081.2/081.9/082/082.0/082.1/082.2/082.3/082.4/082.40/082.41/082.49/082.8/082.9/083/083.1/083.2/083.8/083.9/134.2/136/136.0/136.2/136.21/136.29/136.4/136.5/136.8/136.9/139/139.8/V12.0/V12.00/V12.09 | A75/A75.0/A75.1/A75.2/A75.3/A75.9/A77/A77.0/A77.1/A77.2/A77.3/A77.8/A77.9/A79/A79.0/A79.1/A79.8/A79.9/B20.7/B23.0/B60.1/B60.2/B60.8/B64/B83.4/B88.3/B89/B92/B94.2/B94.8/B94.9/B99/J17.3/L94.6/T61.1 |
| Dermatophytosis | 110.0/110.2/110.6/110.8/110.9 | B35/B35.0/B35.2/B35.8/B35.9/B36.9 |
| Althete's foot | 110.4 | B35.3 |

**Appendix Table 9.** SOFA score calculation

| Organ function | Score | | | | |
| --- | --- | --- | --- | --- | --- |
|  | 0 | 1 | 2 | 3 | 4 |
| Respiratory SOFA | PaO_2_/FiO_2_  $\geq$ 400mmHg | PaO_2_/FiO_2_  $<$ 400mmHg | PaO_2_/FiO_2_  $<$300mmHg | PaO_2_/FiO_2_  $<$ 200mmHg | PaO_2_/FiO_2_  $<$ 100mmHg |
| Cardiovascular  SOFA | MAP $\geq$ 70mmHg | MAP $<$  70 mmHg | Dopamine $<$ 5 or Dobutamine any dose | Dopamine 5.1-15  Or epinephrine $\leq$ 0.1  Or norepinephrine $\leq$ 0.1 | Dopamine $>$15  Or epinephrine $>$ 0.1  Or norepinephrine $>$ 0.1 |
| Liver SOFA | Bilirubin $<$ 1.2 mg/dL | Bilirubin 1.2 – 1.9 mg/dL | Bilirubin 2.0 – 5.9 mg/dL | Bilirubin 6.0 – 11.9 mg/dL | Bilirubin $>$ 12 mg/dL |
| Coagulation SOFA | Platelets $\geq150\times{10}^{3}/\mu L$ | Platelets $<150\times{10}^{3}/\mu L$ | Platelets $<100\times{10}^{3}/\mu L$ | Platelets $<50\times{10}^{3}/\mu L$ | Platelets $<20\times{10}^{3}/\mu L$ |
| Kidney SOFA | Creatinine $<$ 1.2 mg/dL | Creatinine 1.2-1.9 mg/dL | Creatinine 2.0-3.4 mg/dL | Creatinine 3.5-4.9 mg/dL | Creatinine $>$ 5 mg/dL |

**Appendix Figure 1.** SHAP analysis for Sepsis-3 model


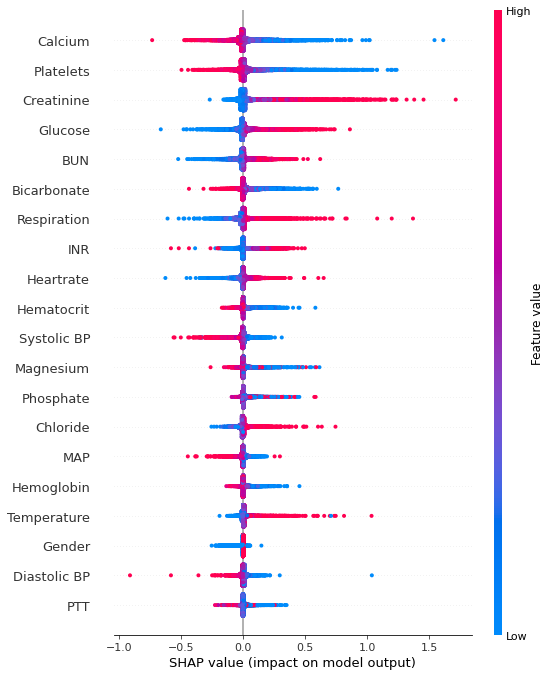


**Appendix Figure 2.** SHAP analysis for ASE model


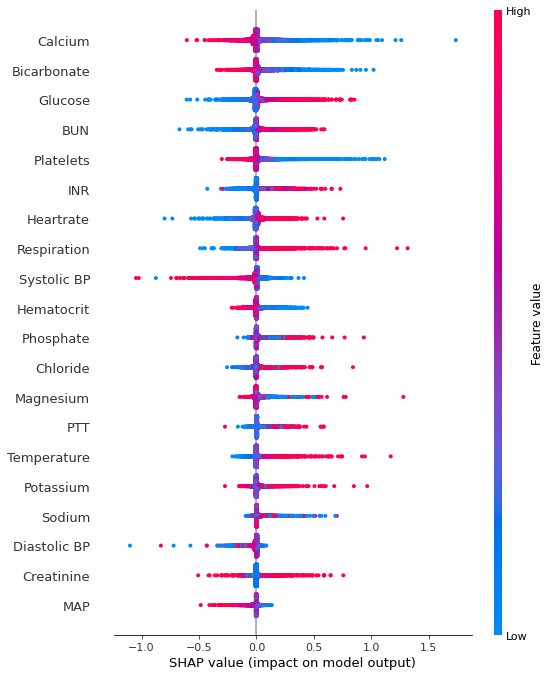

Supplement: Supplementary file 1 [file Data_Sheet_1.docx]
